# Primary Psychoses Among Sentenced Prisoners in Finland

**DOI:** 10.1002/cbm.70034

**Published:** 2026-04-16

**Authors:** Petra Laivonen, Taina Laajasalo, Mika Rautanen

**Affiliations:** ^1^ Faculty of Medicine, Department of Psychology University of Helsinki Helsinki Finland; ^2^ Safety and Protection Unit Finnish Institute for Health and Welfare Helsinki Finland; ^3^ Vanha Vaasa Hospital Vaasa Finland

**Keywords:** comorbidity, corrections, prevalence, prisoner health, prisoners, psychosis, psychotic disorders, solitary confinement

## Abstract

**Background:**

Recent studies suggest an increased prevalence of primary psychotic disorders among sentenced prisoners in Finland. Exploring the extent and correlates of lifetime primary psychoses through high‐quality data is crucial for early identification and effective interventions within correctional settings.

**Aims:**

To establish the current lifetime prevalence and comorbidities of primary psychotic disorders among sentenced prisoners in Finland and to explore associations between these and sex, solitary confinement, forensic mental health examination, index offence and total days imprisoned.

**Methods:**

Data were derived from a subsample (*n* = 295) of the Health and Wellbeing of Prisoners 2023 (Wattu IV) study (*n* = 529). Assessments included Structured Clinical Interview for the US Diagnostic and Statistical Manual for Mental Disorders (DSM‐IV; SCID I and II). Details of offending to date and imprisonment history from official records and self‐reported solitary confinement were included in the database. Logistic regression analysis was used to examine associations between lifetime primary psychotic disorders and variables significantly related in binary analyses.

**Results:**

Twenty‐four (8%) participants had a lifetime primary psychotic disorder, according to SCID‐based data. A further 100 (34%) had a lifetime substance‐induced psychotic disorder. The odds of primary psychotic disorder were over five times (OR 5.22) higher among women than men. For more detailed analyses, prisoners with a drug‐related psychosis could not be separated from prisoners without psychosis. The odds of disciplinary solitary confinement were over three times (OR 3.36) higher among those with lifetime primary psychosis than among all other sentenced prisoners and over four and a half times (OR 4.61) higher among those requesting it. Neither index offence nor total lifetime days imprisoned had any relationship with primary psychosis.

**Conclusions:**

This study updates the understanding of lifetime primary psychotic disorders and their correlates in Finnish prisons, descriptively suggesting a likely rise in their prevalence from 6% in 2017 to 8% in 2023, a 33% increase. The finding that twice as many female as male prisoners have primary psychosis aligns with previous research. The higher use of solitary confinement among sentenced prisoners with lifetime primary psychosis than among other sentenced prisoners, including those with substance‐related psychosis, is new and troubling. As only one third of these prisoners with primary psychosis had had a court‐ordered forensic mental health examination to determine the need for psychiatric treatment and, specifically, compulsory treatment instead of a prison sentence, our findings suggest an urgent case for re‐examining pathways into healthcare for prisoners both during pretrial detention and after sentencing.

## 1 Introduction

Prisoners are disproportionately affected by mental disorders, including severe conditions such as psychotic disorders (Baranyi et al. 2019; Bukten et al. 2024). Likewise, it is widely known that comorbidity of mental disorders is common among prisoners (Baranyi et al. 2022; Mundt and Baranyi 2020).

Prisoners with severe mental disorders have a higher risk of suicide than others, and they are also more prone than others to use violence and to become victims of violence themselves (Baillargeon et al. 2009; Fazel et al. 2016). They are also more likely than others to be subjected to solitary confinement (Dellazizzo et al. 2020), and solitary confinement appears to hinder improvement in mental state (Walker et al. 2014).

Although the incarceration rate had been decreasing in the Nordic countries, it has been increasing in this decade (e.g., for Sweden: Brottsförebyggande rådet 2025; Prison and Probation Service of Finland 2025), and an increasing proportion of those entering prison have a diagnosed mental disorder (Bukten et al. 2024). Further, the proportion with psychosis specifically has increased, as has the proportion of individuals entering prison with a mental disorder and comorbid substance use disorder(s) (Bukten et al. 2024). In Nordic prisons, the prevalence of such mental disorders is higher among women in prison than men (Bukten et al. 2024).

In Finland, Jüriloo et al. (2017) reported a tenfold increase in primary psychosis cases between 2005 and 2016 in Finnish prisons, nearly 6% of prisoners were diagnosed as having a psychotic disorder that was not induced by substance abuse. Further, the proportion of prisoners released to out‐of‐prison care for chronic psychosis is increasing (Lauerma 2021), although taking Jüriloo et al. (2017) together with studies by Joukamaa et al. (2010), it seems that the in‐prison prevalence of primary psychosis in Finland has remained about the same at 6%. Differences in study design may, though, account for some of this seeming discrepancy.

In Finland, forensic mental health examinations have become less likely since the 1990s (Lappi‐Seppälä and Kolehmainen 2021). Further, since the 1980s, treatment of mental disorders in general has shifted from inpatient towards outpatient care (Ahlgrén‐Rimpiläinen 2021). Some have argued that the reduced number of psychiatric beds has led to severely ill patients ending up in prison or even seeking imprisonment purposely to access treatment (Ahlgrén‐Rimpiläinen 2021). Some have even raised concerns that a possible rise in the prevalence of psychosis in prisons indicates that treatment for severe mental disorder may only be accessible through the prison system (Jüriloo et al. 2017; Rautanen and Lauerma 2011). In Finland, a psychiatric prison hospital is designed for acute treatment and care rather than longer‐term treatment and rehabilitation (Ahlgrén‐Rimpiläinen 2021).

Our aim was to examine the prevalence of lifetime primary psychosis as defined by DSM‐IV‐TR (SCID‐I interview, Finnish translation 2008; comparable to F20–F29 in ICD‐10 coding) among sentenced prisoners in Finland, together with comorbidity of substance use disorders and of personality disorders. Substance‐induced psychoses are rated separately, as they differ from primary psychosis in both onset and characteristics. In addition, we investigate whether sex, disciplinary and/or self‐requested solitary confinement, violent crime as the index offence, participation in forensic mental health examination and total lifetime days imprisoned are linked to lifetime primary psychotic disorders among such prisoners.

## 2 Methods

### 2.1 Ethics

Ethical approval for the study was obtained from the Ethics Committee of the Hospital District of Southwest Finland (ETMK 85/1801/2019).

### 2.2 The Wattu IV Study

We used data from the Health and Wellbeing of Prisoners 2023 (Wattu IV) study, a health examination conducted by the Finnish Institute for Health and Welfare, the Prison and Probation Service of Finland and the Health Care Services for Prisoners. Data collection was undertaken with the aim of improving the health of prisoners and supporting service development for such people.

Data were collected between November 2020 and December 2022. Participants were Finnish nationals who had at least one month remaining on their prison sentence. They were interviewed in Finnish, in eight of the 30 prisons in Finland. The eight included four closed and four open prisons. The original protocol required random sampling, but because of COVID‐19 restrictions, this had to be relaxed into a mix of random and convenience sampling. Regardless of this, the prison‐related backgrounds, as defined by number of imprisonments and age at the start of the index imprisonment, were comparable to the whole population of people sentenced to imprisonment in Finland.

The aim of Wattu IV was a comprehensive assessment of the health status of sentenced prisoners in Finland, covering mental, somatic and oral health, addictions, infectious diseases and social relationships. Data were drawn from questionnaires, interviews, health examinations, laboratory tests and oral X‐rays. The methodology and questions were adapted from Borodulin and Sääksjärvi (2019), enabling comparisons with the health status of the general population. Participants provided informed consent for a 20‐year longitudinal follow‐up. A more thorough description of the study is published elsewhere (Rautanen et al. 2024).

The Wattu IV study sample consists of 403 men and 126 women who gave their informed written consent to participate. A minority (17.5%) of those invited refused to participate (response rate 82.5%). The 529 respondents constitute nearly one quarter of the average daily number of sentenced prisoners in Finland (2,187) in the middle of the data collection in 2021 (Prison and Probation Service of Finland 2022). The mean (SD) age for male participants was 37.0 (11.0) years, and for females participants, it was 37.4 (9.8) years.

All participants completed a questionnaire privately, which was reviewed with a study nurse to clarify and manage missing data. Over half of the sample was also assessed by SCID‐I–II interviews (*n* = 295), performed individually by clinicians with SCID training. This subgroup of 295 formed the sample for the study reported here.

### 2.3 Study Variables

Variables of interest included sex, lifetime psychotic disorders, substance use disorders, personality disorders, violent crime as the index offence, disciplinary and self‐requested solitary confinement experiences, participation in forensic mental health examination (a court‐ordered pretrial assessment of responsibility in selected crime cases only) provided by the Health Care Services for Prisoners and total lifetime days imprisoned, with pretrial imprisonment included.

Sex was dichotomously categorised as male or female. Lifetime psychosis, substance use disorders and personality disorders were categorised according to the presence or absence of diagnostic criteria according to DSM‐IV. Violent crime as the index offence refers to the most serious offence for which the individual received the sentence during the study period and was similarly dichotomously categorised as yes or no. Disciplinary and self‐requested solitary confinement experiences were self‐reported and similarly dichotomised, as was participation in forensic mental health examination by the Health Care Services for Prisoners. Information regarding total lifetime days imprisoned up to the time of assessment was provided by the Prison and Probation Service of Finland.

Descriptive analyses included cross‐tabulation and chi‐squared tests. In cases of expected cell count < 5, Fisher's exact test was used. Binary logistic regression was used to examine the independence or otherwise, with lifetime psychosis as the dependent variable. Three logistic regression models were tested, with the final model retaining only the variables significant at the bivariate level. Model selection was informed by the Akaike information criterion (AIC) and Bayesian IC, with lower values indicating better fit. Model fit was assessed using the Hosmer–Lemeshow test (*p* > 0.05 suggests good fit) and pseudo‐*R* ^2^. Statistical analyses were conducted using SPSS Statistics version 28.

## 3 Results

### 3.1 General Description of the Data

239 (81%) study participants were men and 59 (19%) were women. Around a quarter of respondents were under 30 years old (81), and 15% (*n* = 44) were aged 47 years or older.

### 3.2 Lifetime Psychosis

Twenty‐four (8%) participants had a lifetime diagnosis of a primary psychosis according to SCID‐based interview and DSM‐IV criteria equivalent to F20–F29 in ICD‐10 coding (hereafter referred to as the ‘primary psychosis group’). The most common primary psychosis was schizophrenia (*n* = 8), and the second most common was delusional disorder (*n* = 6). An additional seven (2%) had active primary psychosis at the time of study assessment. In addition, lifetime substance‐related psychosis was found in 100 (34%) participants. For all subsequent analyses, these were included in the nonpsychotic comparison group.

The prevalence of lifetime primary psychosis was twice as high among women (14%) as among men (7%). The difference was significant for schizophrenia specifically (*p* = 0.05), but not for any other primary psychosis, nor for substance‐induced psychosis (for details, see Table 1).

**TABLE 1:** Prevalence of lifetime primary psychotic disorders and substance‐induced psychotic disorder among sentenced prisoners in Finland, by sex (*n* = 295).

	Male (*n* = 239)	Female (*n* = 56)	Total (*n* = 295)	*p*	*Φ*
Lifetime					
Schizophrenia	4 (2%)	4 (7%)	8 (3%)	0.05^*^^,^ ^a^	0.13
Schizophreniform disorder	0 (0%)	0 (0%)	0 (0%)	—	—
Schizoaffective disorder	2 (1%)	0 (0%)	2 (1%)	1.00^a^	−0.04
Delusional disorder	4 (2%)	2 (4%)	6 (2%)	0.32^a^	0.05
Acute and transient psychotic disorder	1 (0%)	1 (2%)	2 (1%)	0.34^a^	0.07
Psychosis due to a known physiological condition	3 (1%)	0 (0%)	3 (1%)	1.00^a^	−0.05
Unspecified	2 (1%)	1 (2%)	3 (1%)	0.47^a^	0.04
Any primary psychotic disorder					
Lifetime	16 (7%)	8 (14%)	24 (8%)	0.06	0.11
Acute	5 (2%)	2 (4%)	7 (2%)	0.62^a^	−0.04
Substance‐induced psychotic disorder					
Lifetime	84 (35%)	16 (29%)	100 (34%)	0.35	−0.05
Acute	2 (1%)	0 (0%)	2 (1%)	1.00^a^	−0.04

Abbreviation: *Φ* = phi coefficient, effect size.

^a^ Fisher's exact test was used.

*p ≤ 0.05. **p ≤ 0.01. ***p ≤ 0.001.

### 3.3 Comorbid Substance Use Disorders and Personality Disorders

Almost all (96%) of the prisoners in the primary psychosis group had a comorbid lifetime substance use disorder. Similarly, nearly all participants (96%) in this group had a comorbid personality disorder. In every case of comorbid personality disorder, an antisocial personality disorder was diagnosed, but some individuals also met the criteria for additional personality disorders. Nine in ten (88%) in the primary psychosis group had both comorbid substance use disorder and antisocial personality disorder. One in five (21%) had also had a history of substance‐induced psychotic disorder. For all 16 men and six of the eight women, alcohol was a problem, the sex difference here being significant. Table 2 shows the full picture.

**TABLE 2:** Comorbid lifetime substance use disorders, personality disorders and substance‐induced psychotic disorders among sentenced prisoners with lifetime primary psychotic disorder, by sex (*n* = 24).

	Male (*n* = 16)	Female (*n* = 8)	Total (*n* = 24)	*p*	*Φ*
Comorbid substance use disorder	16 (100%)	7 (88%)	23 (96%)	0.15	−0.30
Alcohol	16 (100%)	6 (86%)	22 (96%)	0.04*	−0.43
Other	15 (94%)	7 (88%)	22 (96%)	0.60	−0.11
Comorbid personality disorder	15 (94%)	6 (75%)	21 (96%)^a^	0.13	−0.32
Antisocial	15 (100%)	6 (100%)	21 (100%)	0.13	−0.32
Other	11 (73%)	6 (100%)	17 (81%)	0.52	0.14
Comorbid substance use disorder and personality disorder	15 (94%)	6 (75%)	21 (88%)	0.29	0.32
Comorbid substance‐induced psychotic disorder	4 (25%)	1 (13%)	5 (21%)	0.48^b^	−0.15

Abbreviation: *Φ* = phi coefficient, effect size.

^a^ *n* = 22.

^b^ Fisher's exact test was used.

*p ≤ 0.05. **p ≤ 0.01. ***p ≤ 0.001.

### 3.4 Violent Crime as the Index Offence

Three quarters (75%) of the prisoners with lifetime primary psychosis had a violent crime as their index offence. There was no significant difference between the primary psychosis group and other prisoners in this respect.

### 3.5 Disciplinary and Self‐Requested Solitary Confinement

Four in five (78%) of the prisoners with lifetime primary psychosis had had at least one experience of solitary confinement for disciplinary reasons, and almost half (48%) had requested it and been held in solitary for this reason. Almost two in five (38%) had experienced both disciplinary and self‐requested solitary confinement, two in five (38%) only disciplinary and 8% only self‐requested solitary confinement. Only one in ten (13%) of the primary psychosis group had had no experience at all of such confinement. Compared with all other prisoners (including those who had had a substance‐related psychosis), significantly more of those with primary psychosis had had at least one disciplinary solitary confinement (*p* = 0.02). This difference was even more striking for self‐requested solitary confinement (*p* ≤ 0.001; see also Table 3).

**TABLE 3:** Prevalence of solitary confinement experiences, violent crime as the index offence, conducted forensic mental health examinations and total lifetime days imprisoned among sentenced prisoners in Finland (*n* = 295).

	Lifetime primary psychotic disorder				
	Yes (*n* = 24)	No (*n* = 271)	In total (*n* = 295)	*p*	*Φ*
Disciplinary solitary confinement	18 (78%)^a^	141 (53%)^b^	159 (55%)^c^	0.02^*^	0.14
Self‐requested solitary confinement	11 (48%)^a^	45 (17%)^b^	56 (19%)^c^	< 0.001^***^	0.21
Violent crime as the index offence	18 (75%)	156 (58%)	174 (59%)	0.10	0.10
Total lifetime days imprisoned	*M* = 1532, SD = 1621	*M* = 1983, SD = 1933	*M* = 1947, SD = 1911	0.27	0.24 (d)
Forensic mental health examination	7 (29%)	49 (18%)	56 (19%)	0.18	0.08

Abbreviations: *Φ* = phi coefficient; *d* = Cohen's *d*.

^a^ *n* = 23.

^b^ *n* = 265.

^c^ *n* = 288.

*p ≤ 0.05. **p ≤ 0.01. *** *p* ≤ 0.001.

Just under a third (29%) of prisoners with lifetime primary psychosis reported having had a forensic mental health examination provided by the Health Care Services for Prisoners. In this respect, there was no statistically significant difference between those in the primary psychosis group and the others (see also Table 3).

### 3.6 Total Lifetime Days Imprisoned

The average total lifetime days imprisoned by the time of the study was 1947 days (SD = 1911, range 19–11,944). Only 15 (5%) had served this time as a continuous period of imprisonment. The average length of current imprisonment was 1125 days (SD = 789, range 79–4730). The observed broad range of incarceration days in this dataset reflects known real‐world variation between first‐time and repeat offenders. There was no statistically significant difference between prisoners in the primary psychosis group and other prisoners in length of imprisonment in these terms.

### 3.7 Multivariate Analysis

We initially tested several logistic regression models, selecting independent variables based on their significant individual associations with the dependent variable (lifetime primary psychotic disorder, substance‐induced psychosis excluded). The final model was chosen for its superior fit statistics (e.g., lowest Akaike information criterion [AIC] and Bayesian IC values) and theoretical relevance. In this model, lifetime primary psychotic disorder was the dependent variable and sex, disciplinary and self‐requested solitary confinement were the independent variables. Because of the low correlation (≤ 0.25) between the independent variables, formal checks for multicollinearity were deemed unnecessary. The pseudo‐*R* ^2^ value of 0.17 indicated that the independent variables explained approximately 17% of the variance in lifetime primary psychosis. The Hosmer–Lemeshow test suggested a good model fit, with a value of 0.44. All independent variables retained their statistically significant relationship (Table 4).

**TABLE 4:** Logistic regression analysis (*n* = 295).

	OR	95% CI	*p*
Sex	5.22	1.79–15.19	0.002^**^
Disciplinary solitary confinement	3.36	1.08–10.42	0.04^*^
Self‐requested solitary confinement	4.61	1.75–12.19	0.002^**^

*Note:* Dependent variable lifetime primary psychotic disorder. Odds ratios (OR), 95% confidence intervals (95% CI) and statistical significance (*p*).

*p ≤ 0.05. **p ≤ 0.01. ***p ≤ 0.001.

The odds of any lifetime primary psychotic disorder were over five times higher among women serving prison sentences than among men (OR = 5.22, 95% CI 1.79–15.19), and the relationships between primary psychotic disorder and both forms of solitary confinement remained significant. Among those with a primary psychotic disorder, the odds of disciplinary solitary confinement were more than three times higher than among those without (OR = 3.36, 95% CI 1.08–10.42), and the odds of self‐requested solitary confinement were more than 4.5 times higher (OR = 4.61, 95% CI 1.75–12.19).

## 4 Discussion

We found that the prevalence of lifetime primary psychotic disorders among sentenced prisoners in Finland is approximately four times higher than in the Finnish general population (Perälä et al. 2007; Suvisaari et al. 2012). In comparison to earlier studies, our figures also suggest a rise in the in‐prison prevalence of lifetime primary psychotic disorders, apparently arising independently of any substance use, from 6% to 8% between 2017 and 2023, suggesting a 33% increase. The current prevalence also seems to be about twice as high as the figure estimated from a systematic review of such studies worldwide (Emilian et al. 2025). There is the possibility that these observed differences may be attributable to methodological variations between studies, but, in Finland, there are plausible drivers for this apparent rise, including the reduction in the number of psychiatric beds, a shift towards outpatient care and fewer forensic mental health evaluations conducted over the past decades (Ahlgrén‐Rimpiläinen 2021; Kaarre et al. 2022).

Our findings highlight, in line with previous studies, the particular vulnerability of women in prison. They also point to a risk of solitary confinement, both disciplinary and self‐requested, among those with a lifetime primary psychotic disorder. The prison environment may exacerbate symptoms of psychotic disorders, which can be misinterpreted as misconduct, whereas some individuals may request solitary confinement to escape stressors. As solitary confinement, whether imposed or self‐requested, has consistently been linked to adverse psychological effects (Haney 2018; Richardson and Lysaker 2023), interventions alleviating symptoms and supporting mental health are essential. It is worth noting, however, that variations in prison systems, penal cultures and solitary confinement policies across countries pose challenges for comparisons between studies (Shalev 2015; Smith 2012).

Although only one in 10 prisoners convicted of violent crime had a lifetime primary psychotic disorder, violent crime was the most common index offence among those in the primary psychosis group. These findings suggest that people with psychosis who are violent may be in danger of being left out of society's support and treatment systems, increasing their risk of ending up in prison. Further, our findings regarding high comorbidity between lifetime primary psychotic disorders, substance use disorders and personality disorders underscore the complexity of prisoners' multimorbidity and the need to consider it in treatment planning.

In Finland, a forensic mental health examination is key to determining a remand prisoner's need for compulsory psychiatric treatment instead of imprisonment, provided that this is raised in the court as part of the assessment of responsibility. Typically, this time‐consuming examination is ordered only in severe violence cases and when psychosis or intellectual disabilities are deemed probable (Seppänen et al. 2020). In 2021, just 44 out of a total of 113 assessed individuals were sent to compulsory treatment instead of a prison sentence (Finnish Institute for Health and Welfare 2025). Our findings suggest that court‐ordered examination is still quite an effective tool for getting individuals in need of involuntary treatment into such treatment, but it may not be in use as often as needed, omitting less severe crime cases and symptoms.

Given that under a third of prisoners with a lifetime primary psychotic disorder had been referred for this detailed expert assessment, questions arise as to whether there are failures of recognition or procedure and whether this could explain at least some of the probable rise in prevalence of primary psychosis in Finnish prisons. Although the high prevalence of antisocial personality disorder among prisoners is generally recognised (Howard and Duggan 2022), the high comorbidity between lifetime primary psychotic disorder and seeming antisocial personality disorder raises the possibility that psychotic disorder may remain hidden under antisocial traits and thus go unnoticed until symptoms become severe.

Future service delivery might be improved by research concentrating on known vulnerable subgroups of prisoners, such as women and those isolated, instead of general prevalence studies. Qualitative methods are needed when trying to understand self‐reported reasons for getting into isolation. It will also be important, in addition, to focus more specifically on prisoners with substance‐induced psychosis for more detailed analysis and to examine, for example, in which ways, if any, they differ from or overlap with prisoners with primary psychotic disorders. A particularly important research stream would be to place emphasis on elucidating the temporal relationships between psychosis and substance use disorders, especially among prisoners, when timing and qualities of incarceration may also play a role.

The much higher rate of use of solitary confinement among sentenced prisoners with lifetime primary psychosis, despite comparable offending and imprisonment histories to those without, raises cause for concern, given the shortcomings in mental health services for prisoners identified across many countries (Forrester et al. 2018). As only a minority of prisoners with lifetime primary psychosis had had specialist forensic mental health examination, and the distribution of having had such examination was unrelated to diagnosis, our findings suggest an urgent case for re‐examining pathways into healthcare for prisoners both during pretrial detention and after sentencing and for reviewing the healthcare needs of sentenced prisoners specifically.

## 5 Limitations

Finland and other Nordic countries have a long history of studying prisoner health (e.g., Joukamaa et al. 2010; Bukten et al. 2024). Unfortunately, differences in study design limit the extent to which findings can be compared within—and indeed outside—Finland. Our results reflect the situation in Finnish prisons at one point between 2020 and 2022 and include only sentenced prisoners. Practical constraints excluded certain important subgroups, such as remand prisoners, foreigners or people with very short sentences. Despite this, the Wattu IV sample proved representative (number of imprisonments and age at start) of the sentenced prisoner population on a given day.

Other limitations include the small size of the primary psychosis group. Further, we were unable to use official healthcare records to ascertain psychiatric diagnoses, and self‐report methods may introduce response bias despite using trained staff for interviewing and scoring. Lastly, participants may have interpreted questions regarding forensic mental health examinations either as general participation or specifically as examinations by the Health Care Services for Prisoners.

## 6 Conclusions

This study updates the understanding of lifetime primary psychotic disorders and their correlates among sentenced prisoners in Finland. The finding that eight in every hundred prisoners have a lifetime primary psychosis is much higher than expected in the general population and suggests a likely increase in prevalence, as compared descriptively with earlier national Finnish prison estimates. Further, prevalence was about twice as high among women in prison as among men in prison. Significantly higher rates of self‐reported experiences of solitary confinement among those with lifetime primary psychosis than among those without are of particular concern, as this hints at treatment insufficiencies, especially as the comparison group included a large number of prisoners who reported at least episodes of substance‐related psychosis. These findings highlight the substantial mental health needs of prisoners with lifetime primary psychosis, the importance of gender‐sensitive approaches and the need to critically re‐evaluate clinical needs in the event of solitary confinement. Strengthening pathways to timely and adequate mental healthcare for sentenced prisoners appears essential. Future research should focus on such pathway evaluation, perhaps for men and women separately, and delineate similarities and differences between substance‐induced psychoses and primary psychotic disorders in prison settings.

## Conflicts of Interest

The authors declare no conflicts of interest.

## Data Availability

The data that support the findings of this study are available from the Finnish Institute for Health and Welfare (THL). Restrictions apply to the availability of these data, which were used under licence for this study. Data are available from the authors with the permission of THL.
